# Typhoid Carrier

**Published:** 1916-03

**Authors:** 


**Typhoid Carrier.** B. R. Wakeman, Sanitary Supervisor of Jefferson, St. Lawrence and N. W. Franklin Counties, N. Y., Health News, Sept. 1915, reports a female domestic, aged 65, who had never had typhoid but had frequently been exposed in the region of "Typhoid Creek." 14 cases were apparently due to her in the last 20 years.

